# Neuromorphic vision with quasi-BICs

**DOI:** 10.1038/s41377-026-02444-w

**Published:** 2026-08-21

**Authors:** Jue Li, Haoye Qin, Qinghua Song

**Affiliations:** Tsinghua Shenzhen International Graduate School, Tsinghua University, Shenzhen, China

**Keywords:** Metamaterials, Nanophotonics and plasmonics

## Abstract

Neuromorphic vision functionalities have been realized by coupling quasi-bound states in the continuum (quasi-BICs) to multiple quantum wells (MQWs). The engineered leaky modes enhance infrared absorption and generate coexisting nonlinear and linear photoresponses that support image preprocessing and in-sensor computing. This approach highlights a new role for quasi-BIC leakage in integrated optoelectronic intelligence.

Biological vision systems perform substantial information processing before signals reach the brain^1^. Within the retina, operations such as contrast enhancement, noise suppression and feature extraction are executed locally, reducing the computational burden of higher visual centers and enabling highly efficient perception. Reproducing such front-end processing capabilities in artificial hardware has become a central objective of neuromorphic vision technologies, which seek to overcome the latency and energy costs associated with conventional von Neumann architectures where sensing and computation are physically separated^2,3^.

Among various optoelectronic platforms, multiple quantum wells (MQWs) provide a promising route for infrared neuromorphic vision systems due to their strong quantum confinement effects, fast carrier dynamics, and compatibility with mature semiconductor fabrication technologies^4–7^. These structures have been widely explored in infrared imaging, optical communication, and sensing applications, making them attractive candidates for in-sensor visual processing^8–10^. However, the optical response of MQWs is fundamentally governed by intersubband selection rules, which require a dominant out-of-plane electric-field component to efficiently drive carrier transitions^11,12^. As a result, the absorption efficiency under normal incidence is intrinsically limited, posing a critical challenge for direct integration into compact imaging systems.

To overcome this limitation, a variety of photonic coupling strategies have been explored. Metallic gratings were introduced to provide the required out-of-plane electric-field component under normal incidence^13^, while plasmonic resonators^14^ and metamaterial absorbers^15^ were subsequently employed to enhance local electromagnetic fields and improve detector responsivity. More recently, metasurfaces have emerged as a versatile platform for tailoring light–matter interactions in MQWs through engineered optical resonances^16,17^. However, most of these approaches primarily focus on enhancing field intensity itself, whereas the momentum-space engineering of radiation channels remains comparatively unexplored.

In parallel, bound states in the continuum (BICs) have emerged as one of the most influential concepts in modern photonics^18–24^. Despite existing within the radiation continuum, these states remain perfectly confined owing to symmetry protection or destructive interference, leading to theoretically infinite quality factors and exceptionally strong light confinement. Over the past decade, BICs and their symmetry-broken counterparts, quasi-BICs (QBICs), have emerged as a powerful platform for manipulating light–matter interactions^25–31^. Most studies of QBICs have focused on maximizing field confinement and minimizing radiation loss. In this context, the radiative leakage generated during symmetry breaking is often regarded as an unavoidable compromise associated with finite quality factors. Yet the emergence of controllable leakage channels raises an intriguing possibility: can radiation leakage itself become a useful resource? Despite extensive investigations of BIC-enhanced light–matter interactions, the potential role of QBIC leaky modes in active optoelectronic functionality and machine-vision hardware has remained largely unexplored.

It is here that the recent work of Zhou et al. makes an important contribution, demonstrating how QBIC-enabled radiation leakage can be systematically accessed and exploited for photodetection functionality^32^. As illustrated in Fig. 1, the authors address this challenge by coupling QBIC metasurfaces to MQWs and exploiting symmetry-breaking-induced leakage modes to enhance intersubband absorption. Their approach transforms radiative leakage from a parasitic loss channel into a functional degree of freedom for engineering photoresponse. By controlling the asymmetry of metallic resonators, the authors continuously tune the transition from a symmetry-protected BIC to a radiative QBIC state, thereby manipulating in-plane momentum components and vertical electric fields within the MQW active region. Numerical simulations reveal that increasing structural asymmetry generates stronger in-plane wave-vector components and enhanced vertical electric fields, which directly improve coupling to MQW intersubband transitions. As a result, the device exhibits a nonlinear increase in photocurrent asymmetrically, while maintaining approximately linear photoresponse characteristics with respect to illumination angle and external bias voltage. This coexistence of nonlinear and linear operating regimes provides multiple functionalities within a single device architecture. Experimental measurements confirm the predicted behavior. Compared with conventional MQW devices, the QBIC-enhanced structures exhibit substantially stronger infrared absorption and photocurrent, achieving more than threefold enhancement under optimized symmetry breaking. Importantly, the enhancement is obtained under normal incidence, directly addressing one of the principal limitations of conventional MQW photodetectors.

**Fig. 1:**
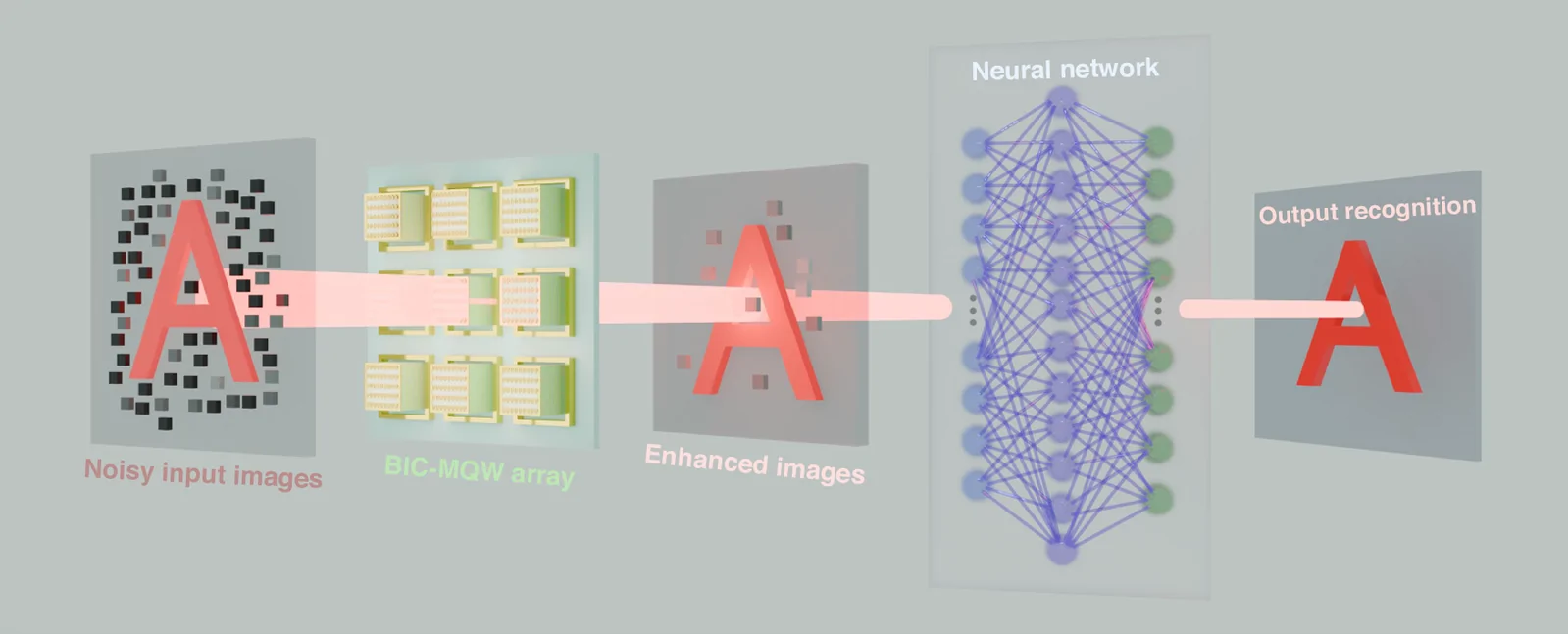
Neuromorphic vision enabled by quasi-BIC-enhanced multiple quantum wells. Noisy optical information is preprocessed by a quasi-BIC-MQW platform before being delivered to a neural network for image recognition, mimicking the hierarchical processing strategy of biological vision systems

Beyond detector performance, the most intriguing aspect of the work lies in its implications for machine vision. The nonlinear photoresponse naturally enables contrast enhancement, amplifying salient image features while suppressing weak background signals. This behavior resembles some of the preprocessing operations carried out in biological visual systems before information reaches higher-level neural circuits. Using this functionality, the authors demonstrate image preprocessing that improves the convergence speed and recognition accuracy of subsequent neural-network inference. Meanwhile, the linear photoresponse characteristics enable hardware implementation of multiply-and-accumulate operations, supporting pattern classification and edge detection directly within the sensing hardware.

More broadly, this work highlights a conceptual shift in how quasi-BICs are viewed. Rather than treating radiative leakage as an undesirable consequence of symmetry breaking, it demonstrates that leakage can serve as a powerful resource for engineering optoelectronic functionality. By bridging photonic resonances, infrared photodetection and neuromorphic computing, the QBIC–MQW platform opens a promising route toward integrated machine-vision systems. Future advances in room-temperature operation, large-scale array integration and on-chip learning architectures may further expand the role of quasi-BICs in intelligent optoelectronic technologies.
